# Heart rate variability changes with respect to time and exercise intensity during heart-rate-controlled steady-state treadmill running

**DOI:** 10.1038/s41598-023-35717-0

**Published:** 2023-05-25

**Authors:** Lars Brockmann, Kenneth J. Hunt

**Affiliations:** rehaLab-The Laboratory for Rehabilitation Engineering, Institute for Human Centred Engineering HuCE, Division of Mechatronics and Systems Engineering, Department of Engineering and Information Technology, Bern University of Applied Sciences, 2501 Biel, Switzerland

**Keywords:** Biomedical engineering, Biomarkers, Cardiology, Control theory

## Abstract

The aim of this work was to investigate the time and exercise intensity dependence of heart rate variability (HRV). Time-dependent, cardiovascular-drift-related increases in heart rate (HR) were inhibited by enforcing a constant heart rate throughout the exercise with a feedback control system. Thirty-two healthy adults performed HR-stabilised treadmill running exercise at two distinct exercise intensity levels. Standard time and frequency domain HRV metrics were computed and served as outcomes. Significant decreases were detected in 8 of the 14 outcomes for the time dependence analysis and in 6 of the 7 outcomes for the exercise intensity dependence analysis (excluding the experimental speed-signal frequency analysis). Furthermore, metrics that have been reported to reach an intensity-dependent near-zero minimum rapidly (usually at moderate intensity) were found to be near constant over time and only barely decreased with intensity. Taken together, these results highlight that HRV generally decreases with time and with exercise intensity. The intensity-related reductions were found to be greater in value and significance compared to the time-related reductions. Additionally, the results indicate that decreases in HRV metrics with time or exercise intensity are only detectable as long as their metric-specific near-zero minimum has not yet been reached.

## Introduction

Heart rate variability (HRV) is the variation in the time interval between consecutive heartbeats (RR intervals—the time elapsed between two successive R waves of the electrocardiogram QRS complex). It is an important physiological indicator related to interactions between the sympathetic and parasympathetic divisions of the autonomic nervous system (ANS)^1,2^. HRV has become an established, non-invasive indicator used across various fields^3–5^. A range of methods for measurement, signal analysis, and interpretation of HRV have been developed and established as formal standards, comprising time domain, frequency domain, and joint time-frequency domain approaches^6–8^. To accurately assess the function of the ANS, time and frequency domain HRV analysis methods are most commonly used^9^. In the frequency domain, four distinct frequency bands have classically been investigated^6^: ultra-low frequency (ULF), with $f<0.00{\overline{3}}$ Hz; very-low frequency (VLF), with $0.00{\overline{3}} \le f <~0.04$ Hz; low frequency (LF), with $0.04 \le f < 0.15$ Hz; and high frequency (HF), with $0.15 \le f \le 0.4$ Hz.

A recent review systematically analysed, among other things, cardiac autonomic responses during exercise using HRV, with a focus on intensity, duration, and modality^10^. It was concluded that the intensity of exercise is the main factor affecting HRV, where a substantial reduction in HRV occurs as intensity increases, and that HRV appears to decrease over time for relatively low-intensity exercise. A distinction could not be made as to whether the decrease in HRV over time was caused by the exercise duration (i.e. time) or by the cardiovascular-drift-related elevated heart rate (HR).

A limitation of the studies included in the review is that only short-duration exercise sessions were studied, implying that only low-frequency (LF) and high-frequency (HF) components of HRV power could be considered. To overcome these limitations, our previous work considered exercise durations that allow analysis of all classical HRV frequency bands including very-low (VLF) and ultra-low frequency (ULF)^11^. Consistent with previous reports^10^, HRV was found to decrease with increasing exercise intensity, and HRV was observed to decrease over time. But the confounding effects of cardiovascular drift on the time-dependent HRV decrease were still at play, and the study concluded that it remains to be clarified whether these changes are due to time or due to increases in HR related to cardiovascular drift^11^.

In the work proposed here, to overcome the drift-related limitations, a feedback control system was employed to keep the heart rate constant throughout the duration of treadmill exercise^12,13^. The technical feasibility of using HR control for the investigation of time-related changes in HRV was proven previously in a pilot study with eight participants^14^; while the outcome measures showed an overall tendency to decrease over time, the decrease was significant ($p < 0.05$) for only seven of the 10 HRV metrics, pointing to the study being statistically underpowered. Further limitations identified in the pilot study, and which are eliminated in the present work, included the employment of a feedback design with low-pass characteristics, thus attenuating information content in the LF and HF bands, and the reconstruction of RR intervals from the HR signal, rather than from direct recordings of raw RR intervals.

By forcing the heart rate to stay constant for the duration of the treadmill exercise, confounding HR-related influences can be removed, and the unobstructed HRV analysis becomes viable. A key challenge with this new HR control approach for HRV analysis is the effect the control loop’s compensating actions can have on specific frequency ranges, potentially affecting frequency-related HRV metrics. Following a suggestion made in our pilot work^14^, a distinct feedback control structure was implemented here to address this limitation by using an input-sensitivity-shaping approach to obtain a uniformly flat frequency response across all frequencies of the treadmill speed signal. Therefore, the treadmill speed signal might be able to act, instead of or as well as the recorded RR intervals, as an indirect source for the frequency-domain HRV analysis, where frequency-specific control loop compensation effects are eliminated. The viability of using speed in this way is explored in the sequel.

The fundamental idea of implementing feedback control of HR to stabilise HR for time-dependency analysis of HRV, as well as the concept of using the treadmill speed signal as a proxy for the RR interval signal to perform an indirect frequency-domain HRV analysis, is new. Aside from our pilot study^14^, this has not been investigated in any previous research.

The aim of this work was to investigate the time and exercise intensity dependence of HRV during steady-state treadmill running while using feedback control to prevent HR drift. Based on the previous findings of Michael et al.^10^ and Hunt et al.^11^, we hypothesised that HRV can be expected to decrease with increasing exercise intensity and throughout the duration of the exercise.

## Methods

### Participants

Thirty-two healthy, regularly exercising (three times per week and at least 30 min per session) adults were included in the study (Table 1; details of an a priori sample size estimate are given later, “Sample size estimate”). Recruitment was carried out by convenience sampling within the Department of Engineering and Information Technology of Bern University of Applied Sciences in Burgdorf and the University of Bern. Of the 32 participants, 29 were male and 3 were female. Smokers and persons with prior history of cardiovascular or respiratory disease or current musculoskeletal complaints or injuries were excluded. Before each test, participants were required to avoid strenuous exercise (24 h), caffeine (12 h), and heavy meals (4 h). The study was approved by the local ethics committee (Ethics Committee of the Swiss Canton of Bern, Ref. 2021-00889), and the participants provided written informed consent prior to participation.

**Table 1:** Participant characteristics, $n = 32$.

	Mean (SD)	Range
Age/year	30.6 (9.2)	22–58
Body mass/kg	76.7 (12.3)	55–105
Height/cm	180.3 (8.6)	160–194
BMI/(kg/m$^2$)	23.5 (2.8)	17.7–29.0

*SD* standard deviation, *BMI* body mass index.

### Feedback control design

**Figure 1:**
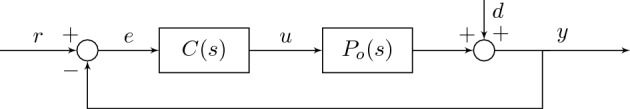
Control structure. *C*(*s*) is the feedback compensator and $P_{o}(s)$ the nominal plant. *r* denotes the reference heart rate, *e* the tracking error, *u* the treadmill speed command signal, *d* a disturbance (mainly heart rate variability), and *y* the actual heart rate.

The control structure was set up as a generic negative feedback control system (Fig. 1) with feedback compensator *C*(*s*) and nominal plant $P_{o}(s)$.

### Control design by input sensitivity shaping

The nominal plant representing the dynamic response between the treadmill speed *u* and the heart rate *y* was defined as a strictly proper transfer function $P_o(s) = B_o(s)/A_o(s)$, constrained in the following to be of first order,

1 $$\begin{aligned} u \rightarrow y \negthickspace : P_{o}(s) = \dfrac{B_{o}(s)}{A_{o}(s)} = \dfrac{k}{\tau s + 1},~n_b < n_a, \end{aligned}$$

where *k* (units beats-per-minute/(m/s)) is the steady-state gain and $\tau$ (units s) is the time constant; $n_b = 0$ and $n_a = 1$ are the degrees of polynomials $B_o$ and $A_o$.

It is known that the plant parameters *k* and $\tau$ vary widely between different people, yet it has been clearly demonstrated that a constant-coefficient, linear, time-invariant compensator based on an average nominal model can deliver stable and accurate HR control performance when applied to a wide range of participants^12–14^, that is to say, robust control is obtained as a consequence of the fundamental ability of feedback to reduce plant uncertainty^15^.

The nominal plant parameters employed here were set to average values obtained from a total of 73 participants from two separate model identification studies, namely $k = 24.88$ and $\tau = 59.28$. These values were derived by combining parameters obtained empirically in two previous studies, more specifically $k = 26.2, \tau = 62.5$ with *n* = 25^16^ and $k = 24.2, \tau = 57.6$ with *n* = 48^17^; the respective sample sizes were taken into account.

The linear feedback compensator, generally described in rational form as $C(s) = G(s)/H(s)$, was intentionally constructed as a merely proper first-order transfer function with an integral term, viz.

2 $$\begin{aligned} e \rightarrow u \negthickspace : C(s) = \dfrac{G(s)}{H(s)} = \dfrac{g_1 s + g_0}{s},~n_g = n_h, \end{aligned}$$

where $n_g=1$ and $n_h=1$ are the degrees of polynomials *G* and *H*. As detailed below, this combination of nominal plant Eq. (1) and compensator Eq. (2) results in a closed-loop characteristic polynomial of degree two and, by virtue of the compensator’s two free parameter $g_1$ and $g_0$, allows arbitrary placement of the two closed-loop poles in the complex plane.

The choice of a proper transfer-function for *C* is to ensure that the input sensitivity function $U_o$ (Eq. (5), below) is also proper and thus remains finite over all frequencies, in line with our design goal to make $|U_o|$ constant for all frequencies (a strictly proper *C* would make $U_o$ strictly proper, whence $|U_o|$ would roll off towards zero as frequency increases).

The closed-loop sensitivity function $S_{o}$, complementary sensitivity function $T_{o}$ and the input sensitivity function $U_{o}$ can be deduced from the control structure^15^. $S_o$ describes the transfer function from disturbance *d* to the controlled output *y* ($d \rightarrow y$), $T_o$ from the reference signal *r* to the controlled output *y* ($r \rightarrow y$), and $U_o$ from the reference signal *r* and the disturbance *d* to the treadmill speed signal *u* ($d \rightarrow u$ and $r \rightarrow u$):

3 $$\begin{aligned} d \rightarrow y \negthickspace : S_{o}(s)&= \frac{1}{1+C P_o} = \frac{A_o H}{A_o H + B_o G}, \end{aligned}$$

4 $$\begin{aligned} r \rightarrow y \negthickspace : T_{o}(s)&= \frac{C P_o}{1+C P_o} = \frac{B_o G}{A_o H + B_o G}, \end{aligned}$$

5 $$\begin{aligned} { d \rightarrow u \; \text {and} \; r \rightarrow u} \negthickspace : U_{o}(s)&= \frac{C}{1+C P_o} = \frac{A_o G}{A_o H + B_o G}. \end{aligned}$$

The closed-loop characteristic polynomial $\Phi$ can be identified as

6 $$\begin{aligned} \Phi = A_o H + B_o G. \end{aligned}$$

The feedback design goal is to shape the input-sensitivity magnitude to be constant across all frequencies. The rationale for this goal is that the treadmill speed command signal *u*, which is linked to HRV disturbance term *d* through $U_o$, might potentially be used for frequency-domain HRV analysis across the whole frequency range.

From Eq. (5), $U_o = A_oG/(A_oH + B_oG)$, and we proceed by taking a cancellation approach to simplify this down to a constant value. As a first step, all plant poles are cancelled by constraining the compensator numerator polynomial *G* to include the known factor $A_o$ and a remaining, unknown factor $G'$ by writing $G=A_oG'$, which results in

7 $$\begin{aligned} U_o = \frac{A_o^2G'}{A_o(H + B_oG')} = \frac{A_oG'}{H + B_oG'}. \end{aligned}$$

Since the plant is assumed strictly proper, $n_b < n_a$, and the compensator proper, $n_g = n_h$, it follows that the order of the characteristic polynomial, Eq. (6), is $n_{\phi } = n_a + n_h$. Since a unique solution of Eq. (6) for *G* and *H* requires that $n_{\phi }$ be equal to the number of free parameters in *G* and *H*, namely $n_h -1 + n_g + 1 = n_h + n_g$, it follows that $n_a + n_h = n_h + n_g \Rightarrow n_g = n_a$ (giving also $n_h = n_a$, since $n_g = n_h$). This in turn implies that, since by construction $G = A_oG'$, the polynomial $G'$ must be of degree zero, i.e. $G'$ is a constant, which we denote $g_0'$. This allows $U_o$ to be written, using Eq. (7), as

8 $$\begin{aligned} U_o = \frac{g_0'A_o}{H + g_0'B_o}. \end{aligned}$$

The next step is to place the remaining closed-loop poles (the roots of $H + g_0'B_o$) at the open-loop pole locations by setting

9 $$\begin{aligned} H + g_0'B_o = A_o. \end{aligned}$$

Substituting in Eq. (8), this results finally, and as desired, in a constant $U_o$,

10 $$\begin{aligned} U_o = g_0', \end{aligned}$$

whereby compensator synthesis amounts to solving Eq. (9) for $g_0'$ and *H*.

Thus far, the derivation was based on the general plant $P_o = B_o/A_o$ and compensator $C = G/H$, but we now specialise the solution to the first order case defined in Eqs. (1) and (2), i.e. using $B_o = k/\tau$, $A_o = s + 1/\tau$, $G = g_1s + g_0$ and $H = s$. With these values, the design equation (9) becomes

11 $$\begin{aligned} s + g_0' \cdot \frac{k}{\tau } = s + \frac{1}{\tau } \end{aligned}$$

giving the solution $g_0' = 1/k$. It follows from Eq. (10) that

12 $$\begin{aligned} U_o = g_0' = \frac{1}{k}. \end{aligned}$$

The various cancellations involved in the derivation also lead to greatly simplified forms for the sensitivity and complementary sensitivity functions in Eqs. (3) and (4), i.e.

13 $$\begin{aligned} S_o(s) = \frac{H}{A_o} = \frac{s}{s + \frac{1}{\tau }} = \frac{\tau s}{\tau s + 1} \end{aligned}$$

and

14 $$\begin{aligned} T_o(s) = \frac{g_0'B_o}{A_o} = \frac{\frac{1}{\tau }}{s + \frac{1}{\tau }} = \frac{1}{\tau s + 1}. \end{aligned}$$

These expressions show that both $S_o$ and $T_o$ have the same bandwidth as the open-loop plant $P_o$ (cf. Eq. (1)), and, indeed, that the closed-loop transfer function $T_o$ is the same as the open loop $P_o$ (up to the scaling factor 1/*k*): these observations point to the “neutrality” of a compensation strategy that achieves a constant input sensitivity function of magnitude equal to the inverse of the steady-state plant gain.

The compensator parameters in $G(s) = g_1 s + g_0$ can be identified by noting $G = g_0'A_o = \frac{1}{k}(s + \frac{1}{\tau })$ to give $g_1 = 1/k$ and $g_0 = 1/(k\tau )$, wherefore

15 $$\begin{aligned} C(s) = \frac{G(s)}{H(s)} = \frac{g_1 s + g_0}{s} = \frac{\frac{1}{k} \left( s + \frac{1}{\tau } \right) }{s} \end{aligned}$$

and it is seen that the compensator parameters depend only on the plant parameters. Using the nominal values $k = 24.88$ and $\tau = 59.28$, the specific compensator used in this study is calculated as

16 $$\begin{aligned} C(s) = \dfrac{0.0402s + 6.780 \times 10^{-4}}{s}. \end{aligned}$$

**Figure 2:**
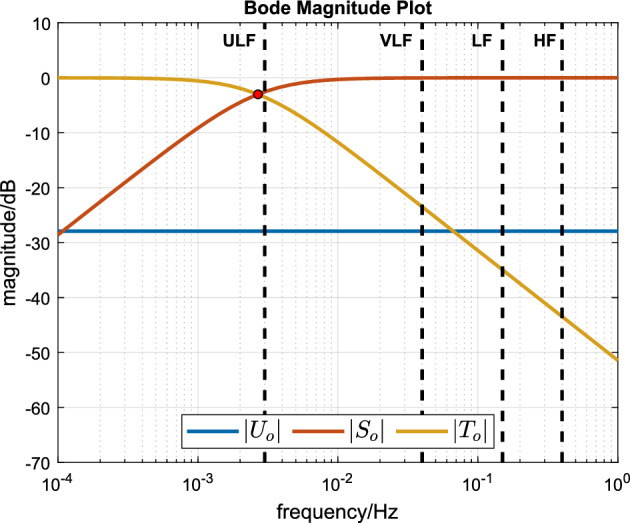
Closed-loop frequency response magnitudes. The sensitivity function is denoted by $S_{o}$, the complementary sensitivity function by $T_{o}$ and input sensitivity function by $U_{o}$. Ultra-low frequency (ULF), very-low frequency (VLF), low frequency (LF), and high frequency (HF) mark distinct frequency bands that have classically been used in heart rate variability analysis. The red dot depicts the $-3~\textrm{dB}$ bandwidth of $S_o$ and $T_o$ (and also of the nominal plant $P_o$).

The sensitivity functions $S_o$, $T_o$ and $U_o$ for this compensator with the nominal plant parameters can be computed using Eqs. (12)–(14) and are plotted in Fig. 2. In particular, it can be seen that $|U_o(j\omega )|$ (blue line) maintains a constant value of 1/*k* over all frequencies ($k = 24.88$ and $20 \log _{10}(1/24.88) = -28$ dB) and that the bandwidth of both $S_o$ and $T_o$ is the same as the open-loop plant bandwidth ($\tau = 59.28$ and $1/(59.28\times 2 \times \pi ) = 0.0027$ Hz). We remark, for the purposes of later discussion, that this bandwidth is just below the upper bound of the ULF frequency band which lies at $0.003{\overline{3}}$ Hz.

### Experimental design and testing protocol

Each participant performed two treadmill running tests at different exercise intensities (lower intensity level 1 [$\mathrm {EIL_{1}}$] and higher intensity level 2 [$\mathrm {EIL_{2}}$]), and each with a duration of 35 min. The intensities for the two tests were set for each participant individually. Each test was carried out on a separate day with at least 48 h between tests. The recording of the two tests ($\mathrm {EIL_{1}}$ and $\mathrm {EIL_{2}}$) was counterbalanced by randomizing the order of employed intensity in order to avoid any order-of-presentation effects. A 10-min warm up was used to assess the HR level and speed at a given baseline exertion level. Participants were asked to choose a running speed equivalent to an exertion level of 13 (i.e. somewhat hard) on the Borg rating of perceived exertion (RPE) scale^18^. The HR recorded during the warm up ($\mathrm {HR_{Warmup}}$) was then averaged and used to calculate the two exercise intensities $\mathrm {EIL_{1}}$ and $\mathrm {EIL_{2}}$. The reference HR for each EIL was calculated as a symmetric deviation from $\mathrm {HR_{Warmup}}$ by $3\%$ of the maximal age-related heart rate ($\mathrm {HR_{max}} = 220-\textrm{age}$) as

17 $$\begin{aligned} \mathrm {HR_{EIL_{1/2}}} = \mathrm {HR_{Warmup}} \pm 3\%\cdot \mathrm {HR_{max}}. \end{aligned}$$

$\mathrm {EIL_{1}}$ corresponds to the lower exercise intensity level and $\mathrm {EIL_{2}}$ to the higher one. Both $\mathrm {EIL_{1}}$ and $\mathrm {EIL_{2}}$ measurements were performed as feedback control tests, where the heart rate was kept constant throughout the running exercise.

### Outcome measures

#### Analysis

Time dependence analysis was performed by calculating and comparing HRV metrics for two consecutive evaluation windows ($w_1$ and $w_2$) with equal duration of 15 min: window $w_1$ was defined from $t = 5~\textrm{min}$ to $t = 20~\textrm{min}$ and window $w_2$ from $t = 20~\textrm{min}$ to $t = 35~\textrm{min}$. For the exercise intensity dependence analysis, HRV metrics were computed for the total duration (denoted $w_1w_2$) of $\mathrm {EIL_{1}}$ and then compared to the HRV metrics computed for the total duration ($w_1w_2$) of $\mathrm {EIL_{2}}$. RR interval outliers or ectopic heartbeats^19^ were identified and removed using an impulse rejection filter^20^ with a threshold value of 7 and a window length calculated to span about 25 s.

#### Time domain HRV metrics

In the time domain, the root-mean-square of successive differences (RMSSD) and the standard deviation of the normal-to-normal (NN) intervals (SDNN) were evaluated and served as primary endpoints. SDNN and RMSSD are defined as follows:

18 $$\begin{aligned} \textrm{SDNN}&= \sqrt{\dfrac{1}{N-1}\sum \nolimits _{i=1}^{N}(\textrm{NN}_{i}-\mathrm {{\overline{NN}}})^2}\end{aligned}$$

19 $$\begin{aligned} \textrm{RMSSD}&= \sqrt{\dfrac{1}{N-1}\sum \nolimits _{i=1}^{N-1}(\textrm{NN}_{i+1}-\textrm{NN}_i)^2}, \end{aligned}$$

where $\textrm{NN}_{i}$ is the *i*th recorded interval and $\mathrm {{\overline{NN}}}$ is the mean value of all $\textrm{NN}$ intervals. *N* is the total number of $\textrm{NN}$ intervals.

#### Frequency domain HRV metrics

In the frequency domain, the average power contained in the frequency bands ULF, VLF, LF, HF, and the total frequency range (TP, Total Power), was computed for the RR signal and the treadmill speed signal using the Lomb-Scargle method for spectral density estimation.

### Statistical analysis

#### Sample size estimate

An a priori statistical power calculation using observed effect size and dispersion from a pilot study with a sample of $n = 8$ participants^14,21^ was performed to obtain a sample-size estimate. Calculations used a significance level of $\alpha = 0.05$ and a required statistical power of $1-\beta = 0.9$. The statistical power calculation was based on the total power of the HRV spectrum (TP, total power spectral density estimate). The pilot study found a difference in paired samples for the log-transformed data of $0.133 \pm 0.236$ (mean ± standard deviation). The power calculation revealed a required sample size of $n = 29$. To cater for the possibility of participants dropping out, a $\sim$10% contingency buffer (+3 participants) was added, resulting in a final sample size of $n = 32$.

#### Analysis

Based on our hypothesis that HRV can be expected to decrease with increasing exercise intensity and throughout the duration of the exercise, one-sided t-tests with significance level $\alpha = 0.05$ were performed for all HRV metrics (time and frequency domain) to assess time and exercise intensity dependence. For the time dependence analysis, HRV metrics computed for $w_1$ were compared to the metrics for $w_2$ (for both intensity levels $\mathrm {EIL_{1}}$ and $\mathrm {EIL_{2}}$). For the exercise intensity dependence analysis, HRV metrics for the total duration of $\mathrm {EIL_{1}}$ were compared to those for the total duration of $\mathrm {EIL_{2}}$. All metrics were log-transformed to reduce skew and make the data conform more closely to the normal distribution. A Shapiro–Wilk test was performed to assess normality, and no data was found to be significantly different from normal.

### Equipment and data collection

All tests were performed on a computer-controlled treadmill (model Pulsar, h/p/cosmos Sports & Medical GmbH, Germany). Treadmill speed was set by a computer running the HR feedback controller within the Simulink Desktop Real-Time environment (The MathWorks, Inc., USA) and communicating with the treadmill over the coscom v3 interface protocol.

Heart rate and raw RR intervals were recorded with a chest-strap-mounted sensor (H10, Polar Electro Oy, Finland) transmitted to an ESP32 development board (Espressif Systems, China) over Bluetooth low energy and sent via serial communication to the control application running on the PC. A Polar V800 wristwatch was employed as a backup method for saving the heart rate and RR intervals. The control application was set up to work with heart rate values transmitted in units of beats per minute (bpm). The V800 and the ESP32 development board saved the more accurate RR intervals, recorded with millisecond resolution, for later analysis.

### Ethical approval

This research was performed in accordance with the Declaration of Helsinki. The study was reviewed and approved by the Ethics Committee of the Swiss Canton of Bern (Ref. 2021-00889). All participants provided written informed consent.

## Results

Sixty-two of the 64 measurements were recorded successfully (one participant dropped out of the study due to muscle pain). Fifty-five of the 62 recorded data sets were able to be used for analysis: three measurements had to be excluded due to insufficient HR control performance, another three due to HR abnormalities and one due to faulty sensor data.

**Figure 3:**
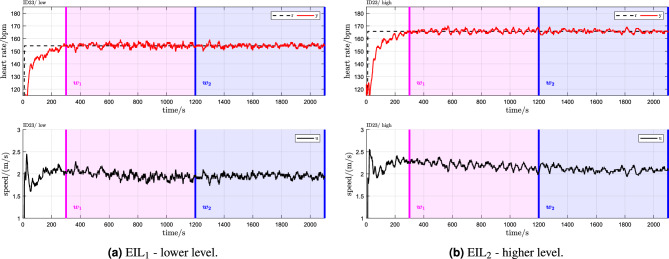
Sample data record for a single participant (P23). Heart rate is denoted by *y*, the constant HR reference by *r* and the treadmill speed command by *u*. The pink and blue shaded areas show the evaluation windows $w_1$ and $w_2$, respectively.

**Figure 4:**
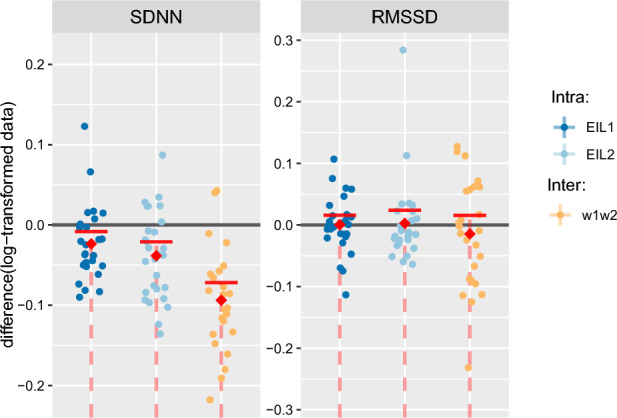
Time domain outcome analysis. Categorical scatter plots of the sample differences, visualising each comparison group for both time domain HRV metrics. Intra-$\mathrm {EIL_{1}}$: $\mathrm {EIL_{1}}$ $w_1$ vs. $\mathrm {EIL_{1}}$ $w_2$; intra-$\mathrm {EIL_{2}}$: $\mathrm {EIL_{2}}$ $w_1$ vs. $\mathrm {EIL_{2}}$ $w_2$; inter-$w_{1}w_{2}$: $\mathrm {EIL_{1}}$ $w_{1}w_{2}$ vs. $\mathrm {EIL_{2}}$ $w_{1}w_{2}$. The red dashed lines are the 95% confidence intervals (CI) with lower bounds at $-\infty$ and red horizontal lines marking the upper bounds. Single-sided t-tests were performed with null hypothesis $H_0\negthickspace :m \ge 0$ and alternative hypothesis $H_0\negthickspace :m < 0$; *m* represents the mean difference and is marked by a red diamond symbol. Significance ($p < 0.05$) corresponds to the value 0 being outwith the 95% CI.

**Figure 5:**
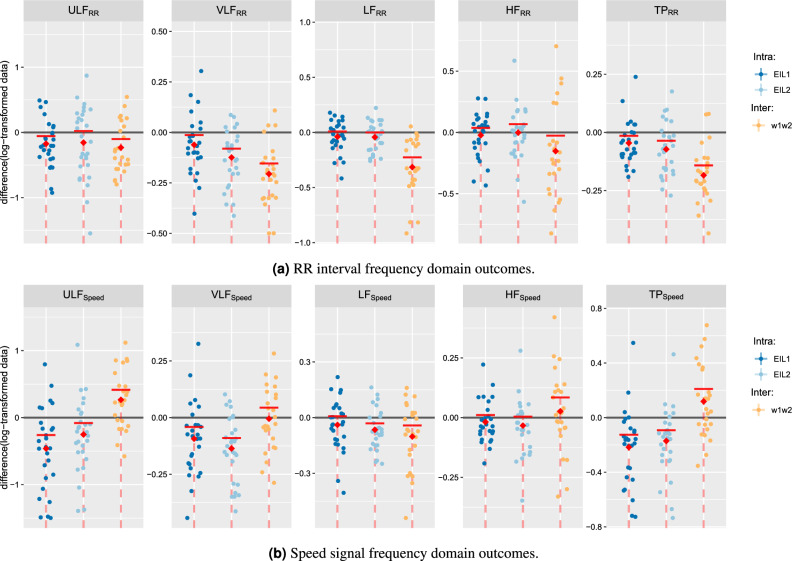
Frequency domain outcome analysis. Categorical scatter plots of the sample differences, visualising each comparison group for all frequency domain HRV metrics: (**a**) for all RR interval derived outcomes; (**b**) for all speed signal derived outcomes; intra-$\mathrm {EIL_{1}}$: $\mathrm {EIL_{1}}$ $w_1$ vs. $\mathrm {EIL_{1}}$ $w_2$; intra-$\mathrm {EIL_{2}}$: $\mathrm {EIL_{2}}$ $w_1$ vs. $\mathrm {EIL_{2}}$ $w_2$; inter-$w_{1}w_{2}$: $\mathrm {EIL_{1}}$ $w_{1}w_{2}$ vs. $\mathrm {EIL_{2}}$ $w_{1}w_{2}$. The red dashed lines are the 95% confidence intervals (CI) with lower bounds at $-\infty$ and red horizontal lines marking the upper bounds. Single-sided t-tests were performed with null hypothesis $H_0\negthickspace :m \ge 0$ and alternative hypothesis $H_0\negthickspace :m < 0$; *m* represents the mean difference and is marked by a red diamond symbol. Significance ($p < 0.05$) corresponds to the value 0 being outwith the 95% CI.

For illustration, a sample data set for a single participant is provided (Fig. 3), while a complete set of categorical scatter plots showing all hypothesis testing results is provided for time domain (Fig. 4) and frequency domain (Fig. 5) outcomes. Table 2 lists the corresponding numerical values.

**Table 2:** Numerical outcomes.

	Time domain HRV metrics		RR-interval frequency domain HRV metrics					Speed signal frequency domain HRV metrics				
	$\textrm{SDNN}$	$\textrm{RMSSD}$	$\mathrm {ULF_{RR}}$	$\mathrm {VLF_{RR}}$	$\mathrm {LF_{RR}}$	$\mathrm {HF_{RR}}$	$\mathrm {TP_{RR}}$	$\mathrm {ULF_{Speed}}$	$\mathrm {VLF_{Speed}}$	$\mathrm {LF_{Speed}}$	$\mathrm {HF_{Speed}}$	$\mathrm {TP_{Speed}}$
Intra												
$$\mathrm {EIL_{1}}$$												
Mean $w_1$	7.79e$-$01	4.93e$-$01	3.31e$-$01	1.37e+00	6.30e$-$01	8.27e$-$02	1.56e+00	− 2.44e+00	− 2.28e+00	− 3.25e+00	− 3.95e+00	− 1.96e+00
sd $w_1$	1.19e$-$01	1.41e$-$01	4.42e$-$01	2.59e$-$01	3.31e$-$01	3.95e$-$01	2.39e$-$01	5.08e$-$01	2.01e$-$01	2.42e$-$01	1.62e$-$01	2.50e$-$01
Mean $w_2$	7.55e$-$01	4.93e$-$01	1.57e$-$01	1.31e+00	5.91e$-$01	5.97e$-$02	1.51e+00	− 2.90e+00	− 2.37e+00	− 3.29e+00	− 3.97e+00	− 2.18e+00
sd $w_2$	1.26e$-$01	1.34e$-$01	4.35e$-$01	3.11e$-$01	3.47e$-$01	4.18e$-$01	2.50e$-$01	5.10e$-$01	2.60e$-$01	2.73e$-$01	1.94e$-$01	2.76e$-$01
MD	− 2.35e$-$02	6.04e$-$04	− 1.74e$-$01	− 6.12e$-$02	− 3.89e$-$02	− 2.30e$-$02	− 4.54e$-$02	− 4.62e$-$01	− 9.32e$-$02	− 3.90e$-$02	− 1.83e$-$02	− 2.17e$-$01
CI	− 8.20e$-$03	1.58e$-$02	− 5.67e$-$02	− 1.30e$-$02	8.66e$-$03	3.69e$-$02	− 1.46e$-$02	− 2.60e$-$01	− 4.10e$-$02	7.46e$-$03	1.09e$-$02	− 1.25e$-$01
*p* value	*7.20e−03*	**5.27e−01**	*8.88e−03*	*1.99e−02*	**8.74e−02**	**2.59e−01**	*9.26e−03*	*2.99e−04*	*2.64e−03*	**8.20e−02**	**1.47e−01**	*2.06e−04*
$$\mathrm {EIL_{2}}$$												
Mean $w_1$	6.97e$-$01	4.83e$-$01	1.13e$-$01	1.20e+00	3.39e$-$01	− 5.57e$-$02	1.40e+00	− 2.34e+00	− 2.30e+00	− 3.34e+00	− 3.91e+00	− 1.94e+00
sd $w_1$	1.36e$-$01	1.28e$-$01	4.59e$-$01	3.32e$-$01	4.29e$-$01	3.25e$-$01	2.71e$-$01	4.26e$-$01	2.40e$-$01	2.80e$-$01	1.25e$-$01	2.28e$-$01
Mean $w_2$	6.59e$-$01	4.86e$-$01	− 4.30e$-$02	1.07e+00	2.96e$-$01	− 5.74e$-$02	1.32e+00	− 2.59e+00	− 2.43e+00	− 3.41e+00	− 3.95e+00	− 2.11e+00
sd $w_2$	1.28e$-$01	1.37e$-$01	5.25e$-$01	3.44e$-$01	4.83e$-$01	3.59e$-$01	2.57e$-$01	5.18e$-$01	2.48e$-$01	3.30e$-$01	1.79e$-$01	2.54e$-$01
MD	− 3.84e$-$02	2.77e$-$03	− 1.56e$-$01	− 1.24e$-$01	− 4.32e$-$02	− 1.76e$-$03	− 7.24e$-$02	− 2.52e$-$01	− 1.36e$-$01	− 6.54e$-$02	− 3.34e$-$02	− 1.71e$-$01
CI	− 2.09e$-$02	2.40e$-$02	2.04e$-$02	− 8.04e$-$02	− 1.62e$-$04	6.75e$-$02	− 3.59e$-$02	− 8.04e$-$02	− 9.02e$-$02	− 3.07e$-$02	4.22e$-$03	− 9.25e$-$02
*p* value	*4.24e−04*	**5.87e−01**	**7.19e−02**	*2.33e−05*	*4.94e−02*	**4.83e−01**	*1.12e−03*	*9.40e−03*	*1.36e−05*	*1.70e−03*	**7.10e−02**	*4.71e−04*
Inter												
$$w_1w_2$$												
Mean $\mathrm {EIL_{1}}$	7.73e$-$01	5.00e$-$01	3.13e$-$01	1.34e+00	6.36e$-$01	8.53e$-$02	1.55e+00	− 2.18e+00	− 2.30e+00	− 3.23e+00	− 3.94e+00	− 1.83e+00
sd $\mathrm {EIL_{1}}$	1.21e$-$01	1.35e$-$01	4.14e$-$01	2.75e$-$01	3.34e$-$01	3.95e$-$01	2.42e$-$01	5.88e$-$01	2.18e$-$01	2.44e$-$01	1.81e$-$01	3.16e$-$01
Mean $\mathrm {EIL_{2}}$	6.79e$-$01	4.86e$-$01	8.03e$-$02	1.14e+00	3.21e$-$01	− 6.77e$-$02	1.36e+00	− 1.91e+00	− 2.31e+00	− 3.34e+00	− 3.91e+00	− 1.71e+00
sd $\mathrm {EIL_{2}}$	1.29e$-$01	1.30e$-$01	4.34e$-$01	3.35e$-$01	4.54e$-$01	2.92e$-$01	2.57e$-$01	4.10e$-$01	2.16e$-$01	2.86e$-$01	1.44e$-$01	2.72e$-$01
MD	− 9.37e$-$02	− 1.45e$-$02	− 2.33e$-$01	− 2.06e$-$01	− 3.15e$-$01	− 1.53e$-$01	− 1.84e$-$01	2.66e$-$01	− 4.80e$-$03	− 1.02e$-$01	2.65e$-$02	1.19e$-$01
CI	− 7.17e$-$02	1.56e$-$02	− 1.02e$-$01	− 1.54e$-$01	− 2.26e$-$01	− 2.63e$-$02	− 1.42e$-$01	4.15e$-$01	4.38e$-$02	− 4.21e$-$02	8.44e$-$02	2.10e$-$01
*p* value	*7.78e−08*	**2.09e−01**	*2.76e−03*	*2.79e−07*	*1.41e−06*	*2.49e−02*	*5.93e−08*	**9.97e−01**	**4.34e−01**	*3.84e−03*	**7.80e−01**	**9.82e−01**

All outcomes are log-transformed, thus dimensionless; *MD* mean difference (intra-group comparison: $w_2 - w_1$; inter-group comparison: $\mathrm {EIL_{2}}$ - $\mathrm {EIL_{1}}$); *CI* upper 95% confidence interval boundary; *p* value: derived from a single-sided t-test performed on the log-transformed data of the respective comparison groups. The *p* values were conditionally emphasized: $p < 0.05 \rightarrow$ italic else bold).

### Time domain outcomes

SDNN was found to significantly decrease over time at $\mathrm {EIL_{1}}$ ($p = 0.007$) and $\mathrm {EIL_{2}}$ ($p < 0.001$), and was observed to be lower at a higher intensity ($p < 0.001$). RMSSD, on the other hand, revealed no significant differences, neither for the intensity comparison ($p = 0.209$) nor the time dependence analysis at $\mathrm {EIL_{1}}$ ($p = 0.527$), nor the time dependence analysis at $\mathrm {EIL_{2}}$ ($p = 0.587$).

### Frequency domain outcomes

The frequency domain outcomes are separated into results derived from the RR interval analysis and results derived from the speed signal analysis (Fig. 5).

For the RR interval frequency domain analysis, all frequency-related HRV metrics (ULF, VLF, LF, HF, and TP) were found to decrease significantly with increasing exercise intensity ($p, \mathrm {ULF_{RR}}=0.003$; $\mathrm {VLF_{RR}}<0.001$; $\mathrm {LF_{RR}}<0.001$; $\mathrm {HF_{RR}}=0.025$; $\mathrm {TP_{RR}}<0.001$). The outcomes for the time dependence analysis at $\mathrm {EIL_{1}}$ showed significant decreases for ULF, VLF, and TP ($p, \mathrm {ULF_{RR}}=0.009$; $\mathrm {VLF_{RR}}<0.020$; $\mathrm {TP_{RR}}=0.009$), moderate evidence for LF ($p, \mathrm {LF_{RR}}=0.087$) and little to no evidence for HF ($p, \mathrm {HF_{RR}}=0.259$). For the time dependence analysis at $\mathrm {EIL_{2}}$, LF, VLF, and TP were observed to decrease significantly ($p, \mathrm {LF_{RR}}=0.049$; $\mathrm {VLF_{RR}}<0.001$; $\mathrm {TP_{RR}}<0.001$). ULF showed moderate evidence of decrease ($p, \mathrm {ULF_{RR}}=0.072$). For HF, no evidence of a decrease was found ($p, \mathrm {HF_{RR}}=0.483$).

For the speed signal frequency domain analysis, ULF, VLF, and TP were found to decrease over time at both $\mathrm {EIL_{1}}$ and $\mathrm {EIL_{2}}$ ($p, \mathrm {ULF_{Speed}}<0.001$; $\mathrm {VLF_{Speed}}=0.003$; $\mathrm {TP_{Speed}}<0.001$ for $\mathrm {EIL_{1}}$ and $p, \mathrm {ULF_{Speed}}=0.009$; $\mathrm {VLF_{Speed}}<0.001$; $\mathrm {TP_{Speed}}<0.001$ for $\mathrm {EIL_{2}}$). A significant difference was found in LF at $\mathrm {EIL_{2}}$ ($p, \mathrm {LF_{Speed}} = 0.002$), and moderate evidence for a decrease was observed in LF at $\mathrm {EIL_{1}}$ ($p, \mathrm {LF_{Speed}} = 0.082$). No significant differences in HF at $\mathrm {EIL_{1}}$ ($p, \mathrm {HF_{Speed}} = 0.147$) and moderate evidence at $\mathrm {EIL_{2}}$ ($p, \mathrm {HF_{Speed}} = 0.071$) were identified. The exercise intensity dependence analysis revealed significant differences in LF and no evidence in ULF, VLF, HF, and TP ($p, \mathrm {ULF_{Speed}}=0.997$; $\mathrm {VLF_{Speed}}=0.434$; $\mathrm {LF_{Speed}}=0.004$; $\mathrm {HF_{Speed}}=0.780$; $\mathrm {TP_{Speed}}=0.982$).

## Discussion

This work aimed to investigate the time and exercise intensity dependence of HRV during steady-state treadmill running while using feedback control to prevent HR drift: we hypothesised that HRV can be expected to decrease with increasing exercise intensity and throughout the duration of the exercise.

Analysing the time dependence of time and frequency domain HRV metrics, $\mathrm {EIL_{1}}$ and $\mathrm {EIL_{2}}$ revealed similar findings. SDNN, as well as ULF, VLF, LF, and TP (all except HF), computed using RR intervals and the treadmill speed signal, in the main showed strong evidence of decreasing in value over time (with moderate evidence for $\mathrm {ULF_{RR}}$ at $\mathrm {EIL_{2}} \rightarrow p = 0.072$; $\mathrm {LF_{RR}}$ at $\mathrm {EIL_{1}} \rightarrow p = 0.087$ and $\mathrm {LF_{Speed}}$ at $\mathrm {EIL_{1}} \rightarrow p = 0.082$). RMSSD and HF (RR and speed) did not significantly decrease over time. Combining these results with the tendency that most HRV measures reach an intensity-dependent near-zero minimum, and that HRV metrics believed to be associated with cardiac parasympathetic activity (i.e. RMSSD and HF) have been reported to reach that minimum more rapidly (usually at moderate intensity)^10^, gives good reason to infer that HRV decreases with time as long as the intensity-dependent near-zero minimum has not yet been reached. This conclusion is also supported by the findings from our pilot study^14^, where exercise performed at light intensity revealed HF and an RMSSD-related metric to decrease over time ($p = 0.053$ for RMSSD proxy; $p = 0.047$ for HF). It would seem that with the low exercise intensity, RMSSD and HF had not yet reached a minimum and thus were able to decrease over the observation interval.

The intensity-level comparison identified a significant decrease in SDNN and in all frequency-domain HRV metrics (computed for the RR intervals) with increasing exercise intensity. RMSSD did not change, indicating the presence of an intensity-dependent near-zero minimum below $\mathrm {EIL_{1}}$. Despite the consistent decrease in frequency-domain HRV metrics computed for the RR intervals, the corresponding outcomes for the speed signal’s frequency-domain HRV metrics differed greatly. VLF and HF stayed approximately constant, while ULF and TP increased. This finding is contrary to expectations considering the connection of the RR interval signal via the controller to the speed signal. With the control loop’s flat input sensitivity function and the consistent control structure across both exercise intensities, intensity-dependent changes in outcomes were expected to be reflected in both the RR intervals and the speed signal. Based on the large increase in ULF power, we suspect this outcome to be affected by an inconsistent plant model. A deviation from the nominal plant can directly affect the actual characteristics of the sensitivity functions. The conspicuous increase in ULF leads us to believe that a decrease in the actual steady-state gain parameter *k* with increasing exercise intensity might have been the cause as the limit of $|U_{o}(j\omega )|$ as frequency tends to 0 is 1/*k* (see “Feedback control design”). A lower than nominal *k* would reduce the control loop’s dampening effect on the impact the RR signal can have on the treadmill speed, thus leading to an overall increase in power. However, this argument may not be in agreement with a previous study^17^, where model plant parameters *k* and $\tau$ were reported not to be significantly dependent on exercise intensity. A second contributory factor might have been the larger speed reduction observed at higher intensities. Throughout a running exercise bout, the treadmill speed typically drifts downwards to compensate for cardiovascular-drift-related HR changes: this downward trend in treadmill speed was observed to be greater at higher intensities.

As a consequence, we suggest that the method of using the treadmill speed signal as a proxy for the RR intervals during heart rate stabilised running exercise, in order to perform frequency-domain HRV analysis where frequency-specific control loop compensation effects are eliminated using input-sensitivity-shaping, needs further investigation to be applicable for an intensity dependence analysis. On the other hand, for the time dependence analysis, this method produced results that closely matched the trends found in frequencies primarily unaffected by the control loop (VLF, LF, and HF) of the original RR interval analysis. This suggests that the speed signal could be used as an alternative to detect trends in frequencies primarily affected by the control (namely ULF) without the influence of the control loop’s compensation effects.

We acknowledge that the duration of 15 min for the analysis windows $w_1$ and $w_2$ is relatively short for the ULF power estimation. Longer analysis windows are generally more desirable but would place additional demands on the participants performing the running exercise, therefore a balance has to be found.

## Conclusion

In summary, feedback control of heart rate was successfully employed to answer the question of whether time-dependent HRV changes occur due to time itself or due to cardiovascular-drift-related heart rate increases. Most HRV metrics were found to decrease with time and with exercise intensity. The exercise-intensity-related reductions were generally found to be greater in value and significance compared to the time-related reductions. HRV metrics that have been reported to reach an intensity-dependent near-zero minimum rapidly (usually at moderate intensity) were found to be near constant over time and only barely decreased with intensity, indicating that decreases in HRV metrics with time or exercise intensity are only detectable as long as their metric-specific near-zero minimum has not yet been reached.

## Data Availability

The raw data supporting the conclusions of this article can be found in the OLOS repository (10.34914/olos:jrpnz3eeh5ephkljdcaieefxqi) and will be made available by the authors on reasonable request.
